# Ultraviolet Fluorescence Photography—Choosing the Correct Filters for Imaging

**DOI:** 10.3390/jimaging8060162

**Published:** 2022-06-07

**Authors:** Jonathan Crowther

**Affiliations:** JMC Scientific Consulting Ltd., Egham, Surrey TW20 8LL, UK; jonathan@jmcscientificconsulting.com

**Keywords:** photography, ultraviolet, fluorescence, imaging, conservation, forensics

## Abstract

Ultraviolet (UV) fluorescence is a valuable tool for the imaging of a wide range of subjects. Like all imaging techniques, the key to success depends on the correct choice of equipment and approach used. In fluorescence photography, a filter is placed in front of the camera lens to block unwanted short-wavelength light from entering the camera, which would compromise the image. However, some filters exhibit fluorescence under UV light and can therefore have the potential to produce a color cast on the image. Filters also vary in how well they block unwanted light. A range of commonly used optical filters was assessed for fluorescence under UV light, and their optical transmission between 250 nm and 800 nm was measured. Finally, a simple method to enable the researcher to determine the fluorescence of the filters that they are using or wish to use for their work is described. The results indicate that the filters tested demonstrated a wide range of fluorescence under UV light and varying degrees of UV blocking. Some filters tested had equivalent or reduced fluorescence compared to Schott KV-418, which is a widely used, but, unfortunately, no longer manufactured UV blocking filter commonly used for fluorescence photography.

## 1. Introduction

Photography is a valuable research tool for the conservator and can even be used to visualize aspects of a subject that are normally impossible to see to the naked eye. One of these techniques is fluorescence photography, where a short-wavelength light, typically ultraviolet (UV), is used to irradiate the sample, and longer-wavelength light is imaged photographically [1,2,3]. Fluorescence photography is used in a wide range of research fields, including art conservation, forensics, healthcare, dermatology and ecology [4,5,6,7,8,9,10,11,12,13,14,15,16,17]. Like all photographic techniques, fluorescence photography has some basic principles that must be adhered to in order to ensure high quality, reproducible results [3]. One of these requirements is to block the wavelengths of light produced by the light source, thereby ensuring the only light being imaged is coming from the fluorescence of the subject rather than reflection [18]. While most digital cameras have some form of internal filtration over the sensor, this cannot always be relied upon to block all the reflected UV light, therefore requiring the use of a filter in front of the camera lens. Moreover, camera lenses themselves can fluoresce under UV light, making it vitally important to block any UV light before it can enter the lens and camera.

A wide range of photographic filters is available to the researcher; however, it is not always clear which one should be used or how they differ in terms of their performance. As well as consideration of the wavelengths they block, some filters themselves exhibit fluorescence under UV light and could therefore produce a visible color cast when used for imaging [18]. For instance, if the subject exhibits areas of specular reflection, UV light would be reflected back to the filter on the lens, and the filter would then fluoresce, emitting visible or infrared (IR) light. One filter was widely reported for use for UV fluorescence photography—Schott KV-418—as it was reported to have either no or very low fluorescence under UV light, in addition to efficiently blocking UV [1,2,3]. However, this filter is no longer manufactured and, as such, is difficult to find. It was also limited in the sizes it was available in, which could have an impact on its use with different lenses. What is needed are other filters available that offer well-defined cutoff wavelengths, good UV blocking and low fluorescence. While some information exists on the fluorescence of photographic filters, it is typically either qualitative [19] or only available for specific technical filters [20] rather than standard photographic filters.

This paper discusses a number of areas that are of importance to the researcher involved with UV fluorescence photography. Firstly, a range of commonly available photographic filters was assessed for fluorescence under UV light, and the degree of fluorescence was quantified using a described method. Filters covering a wide range of cutoff wavelengths were examined. The optical transmission of the test filters between 250 nm and 800 nm was also measured and discussed. Finally, a simple method to enable the researcher to determine the fluorescence of the filters that they are using or wish to use for their work is described.

## 2. Materials and Methods

### 2.1. Methods

A combination of photography and spectroscopy was used to assess the filters analyzed here. UV induced visible light fluorescence photography, using a broad band UV source, was used to determine the degree of fluorescence from a range of photographic filters. Optical transmission spectroscopy was used to measure the filter’s transmission from the UV through to the IR.

### 2.2. Filter Fluorescence Measurement

Filter fluorescence testing consisted of two key elements; a controlled UV light source and an imaging system. A Hamamatsu LC8 source fitted with a 200 W Xenon lamp was used as the light source for testing, which was positioned at a distance of 40 cm from the optical filter being assessed. The light source was filtered using a Baader U-Venus filter (Baader Planetarium GmbH, Mammendorf, Germany) placed over the emission port, and the light was left to warm up and stabilize for at least 30 min prior to any imaging. The filtration resulted in a range of UV wavelengths from 320 nm to 400 nm being used for the fluorescence test. An irradiance spectrum of the light source output was measured using an Ocean Optics FX Spectrometer using a cosine corrector on a 600 µm extreme solarization fiber optic patch cord.

The photographic filter tested was cleaned and placed 40 cm from the light source inside an enclosure that had been painted with Semple Black 2.0 paint (Culture Hustle, Dorset, UK), as this paint was previously shown to be highly light-absorbing from the UV through to the IR [21]. The enclosure was painted in this way to minimize the possibility of any reflected light hitting the filter to be tested or being reflected back towards the camera. Four layers of the paint were applied to the inside of the housing, with approximately 1 h of drying time in between each coat. The fluorescence of the test filter was imaged with a Canon EOS 6D camera, using a Rayfact 105 mm f4.5 UV lens [22], with a 420 nm cut off long pass filter on the lens, which did not show any visible fluorescence under UV (the 420 nm long-pass filter used as the blocking filter on the lens is referred to as the KS 420 nm LP filter in the text). A camera sensitivity of ISO6400 and a lens aperture of f11 were used, and images were captured as 14-bit RAW files and jpegs within the camera using daylight white balance. RAW files were used for analysis, and jpegs were used to enable visual comparison of the fluorescence and to determine its color. All imaging took place in a darkened room, and the LED lights on the front of the light source had black tape applied over them. RAW images were imported into RawDigger x64 Research Edition (LibRaw LLC, Potomac, MD, USA, version 1.2.23) and analyzed as RAW composite files. An area within the image of the fluorescing filter was masked within the RAW file, and the Red, Green 1, Blue and Green 2 channels from the masked area were recorded. All data were recorded, processed and plotted using Microsoft Excel 2010. The responses from the different channels were then averaged to give an average fluorescence response for a given exposure. At least 3 different exposures were imaged for each filter, and the RAW file channel measurements from each of these were then normalized based on exposure time to give a ‘fluorescence value per second’ to enable the filters to be quantitatively compared with each other. The use of RAW file channel data in this manner was shown to be quantitative with regard to the amount of light being collected, enabling direct comparisons to be made between the different fluorescence scores [23].

### 2.3. Filter Transmission Measurement

The filter transmission spectrum was measured between 250 nm and 800 nm using an Ocean Insight DH-2000-BAL light source and FX spectrometer, using 600 µm extreme solarization fiber optic patch cords for the input and output fibers, along with 74-UV collimating lenses, all mounted in an Ocean Insight RTL-T stand. Transmission of the filters was carried out with both the Deuterium light alone and the Deuterium and Halogen lights combined. The data from both scans were then combined in order to minimize the effects of stray light within the spectrometer.

### 2.4. Imaging of Vase under UV Light

A glass vase was imaged under UV illumination (lighting was as described in Section 2.1, with the vase being placed at a distance of 40 cm from the light source). The images were captured using a Canon EOS 6D camera. Two lenses were tested; a Rayfact 105 mm f4.5 UV lens and a Micro Nikkor 105 mm f4. Images were captured with no filter on the lens and with either the KS 420 nm LP, Firecrest UV400, Quaser 415 nm 1%, or a LaLaU UV pass filter. The LaLa U UV pass filter (UVIROptics, Eugene, OR, USA) blocks visible and IR light while letting UV pass. Camera settings and image analysis were as described in Section 2.2, and all exposures were 1/2 s.

## 3. Results and Discussion

A wide selection of photographic filters is available to the researcher to help with selecting which wavelengths they wish to image, ranging from technical glass such as Schott GG400 and GG420 to more consumer-focused colored lens filters such as Hoya and Tiffen yellow filters. However, determining which can be useful for use in fluorescence imaging can be difficult. In the work described here, a range of filters from different sources and with different cutoff wavelengths from the visible through to the Infrared (IR) were tested for fluorescence under UV light and also had their optical transmission spectra measured. The filters tested have uses in various applications but were selected as they all block the shorter wavelength UV light. Moreover, a method for the researcher to test their own filters for UV-induced visible fluorescence was also described.

**Figure 1:**
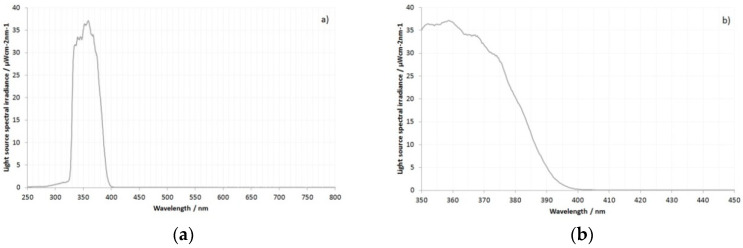
Output of the test light source after filtering with Baader U-Venus filter: (**a**) Full spectral range of 250 nm to 800 nm; (**b**) 350 nm to 450 nm.

In order to control the illumination of the test filters, a broadband continuous UV light source based on a Xenon lamp was used. The output of the light source after filtering with the Baader U-Venus filter is shown in Figure 1. The light source spectrum was measured at the same distance that the filters were tested.

The Baader U-Venus filter provides strong out-of-band blocking in the visible and IR regions [23], and the resultant illuminant light is comprised of UV between 320 nm and 400 nm. The small shoulder on the irradiance curve at 280 nm and 320 nm is due to the effects of stray light in the spectrometer rather than transmission light by the filter and is sometimes seen with spectra from solid-state spectrometers, especially when dealing with filters with sharp cutoffs [24]. As shown in Figure 1, the light source irradiance peaked at around 37 µWcm^−2^ nm^−1^ at about 360 nm, which is similar to the intensity of UV light in sunlight at sea level, as reported in [25]. As such, UV damage to the filters during the tests described here was not expected.

**Figure 2:**
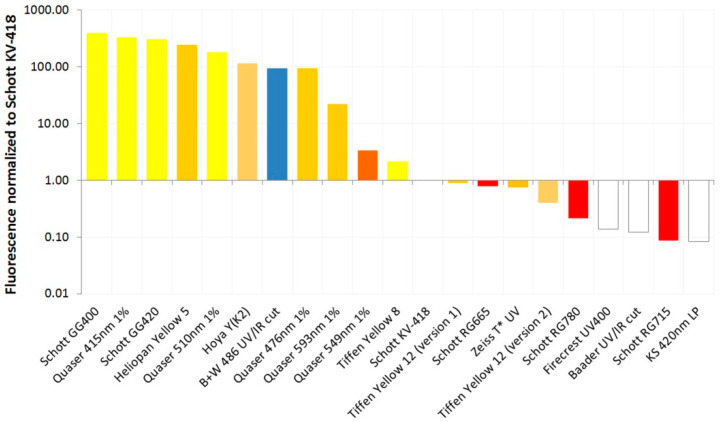
Fluorescence of the test photographic filters normalized to Schott KV-418. Bar color indicates the color of the filter fluorescence.

The test filters exhibited a wide range of fluorescence under UV light, both in terms of the brightness of the fluorescence and its color. The relative degree of fluorescence (normalized for the exposure times used during image capture) and its color are given in Table 1, along with the filter thicknesses. The filter fluorescence ranked and normalized in relation to Schott KV-418 is shown in Figure 2 to enable the filters to be more readily compared.

It should be noted that the fluorescence in Figure 2 is shown as a logarithmic scale because there is such a wide range of fluorescence in the test filters. Some filters, such as the Schott GG400 and GG420; the Quaser 415 nm, 476 nm and 510 nm; the Heliopan Yellow 5; and Hoya Yellow Y(K2), exhibited extremely strong fluorescence under UV light, while others, such as Schott KV-418, Baader UV/IR cut, Tiffen Yellow 12, the RG series IR filters, Firecrest UV400 and Zeiss T* UV, showed very little if any visible fluorescence. There were almost four orders of magnitude differences in the degree of fluorescence between the filters tested. The Quaser filters (sometimes reported with the spelling Quasar) seem to be long pass filters similar to the Schott OG and GG range and were part of a range of forensic imaging filters sold by Mason Vactron [26]. Schott KV-418, which is commonly used as a UV blocking filter in fluorescence imaging, exhibited a very low degree of fluorescence; however, a number of the filters tested here showed similar or even lower fluorescence.

The majority of the filters that fluoresced did so as yellow or orange (or pale red in the case of the RG filters). The B + W 486 UV/IR blocking filter fluoresced blue, though, and it was the only filter tested that showed this color. This could be due to the dichroic coating present on the filter. This behavior was very different from the other UV/IR blocking filter tested here, the Baader UV/IR filter, which did not show any significant fluorescence but also had a dichroic coating present on it. This suggests that the nature of any dichroic coating present could have a significant impact on the filter fluorescence properties. The IR filters (RG665, 715 and 780) showed a pale red fluorescence, which was less intense than that observed for Schott KV-418.

Assessment of the images showed that the Semple Black 2.0 paint, which was previously shown to have very low UV reflectance by analysis of its diffuse reflectance [21], also exhibited a very low degree of fluorescence under UV, making it an ideal black paint for use in making enclosures for this type of work.

**Figure 3:**
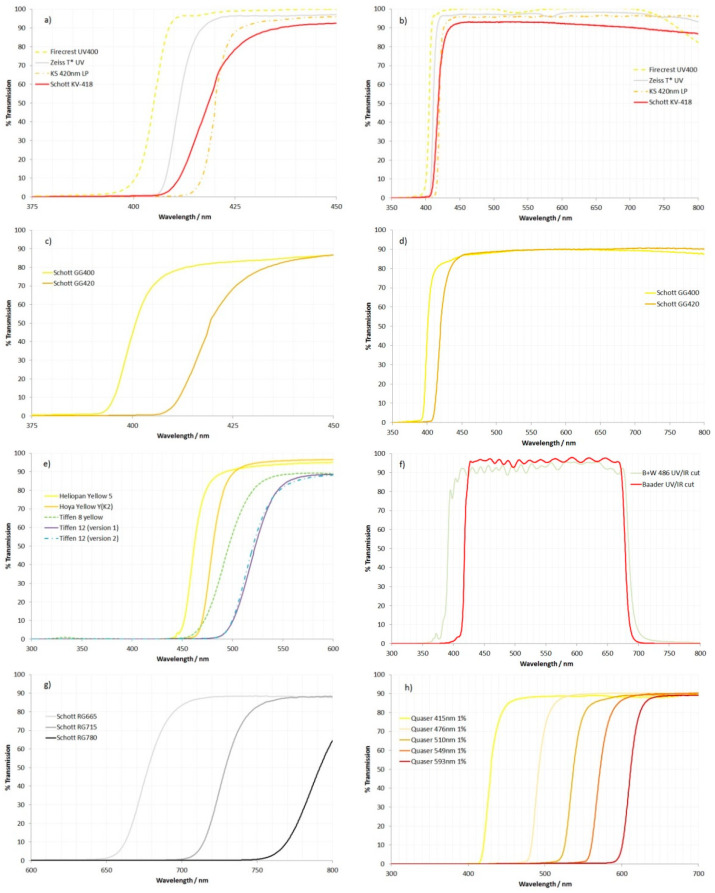
Optical transmission spectra of the test filters: (**a**) Firecrest UV400, Schott KV-418, Zeiss T* UV and KS 420 nm LP from 375 nm to 450 nm; (**b**) Firecrest UV400, Schott KV-418, Zeiss T* UV and KS 420 nm LP extended range from 350 nm to 800 nm; (**c**) Schott GG400 and GG420 from 375 nm to 450 nm; (**d**) Schott GG400 and GG420 from 350 nm to 800 nm; (**e**) Heliopan Yellow 5, Hoya Yellow Y(K2), Tiffen Yellow 8, Tiffen Yellow 12 (version 1 and 2) from 300 nm to 600 nm; (**f**) B + W 486 UV/IR cut and Baader UV/IR cut from 300 nm to 800 nm; (**g**) Schott RG665, RG715 and RG780 from 600 nm to 800 nm; (**h**) Quaser 415 nm 1%, 476 nm 1%, 510 nm 1%, 549 nm 1%, 593 nm 1% from 300 nm to 700 nm.

While the region of 250 nm to 800 nm was scanned for all the filters, the curves shown in Figure 3 were split into groups with similar properties, and the wavelength ranges displayed were chosen to concentrate on the areas where the optical properties of the filters changed.

The Schott KV-418 filter shows a gradual cutoff starting at about 405 nm (Figure 3a) and then rising through 50% maximum transmission at approximately 418 nm as expected. It then remained relatively flat until around 580 nm, after which it gradually reduced to 800 nm (Figure 3b). The rate at which its transmission rises is similar to the Schott GG400 and GG420 (Figure 3c,d) but slower than that seen for the Zeiss T* UV filter and Firecrest UV400 filter (Figure 3a).

Of the five yellow/orange standard photographic filters shown in Figure 3e, the Tiffen 8 and 12 filters showed a less well-defined cutoff in the transmission than the Hoya and Heliopan yellow filters. Moreover, the Tiffen 8 yellow filter did not completely block the UV light, showing a small but observable transmission peak at around 340 nm. The transmission spectra for the two different versions of the Tiffen 12 filter were almost identical to each other.

The two UV/IR cut filters shown in Figure 3f also behave differently from each other. The B + W 486 filter lets in a significant amount of UV light below 400 nm (as far down as 360 nm) and IR light above 700 nm. The Baader UV/IR cut filter was much more efficient at blocking UV and IR, however, and has been reported previously for its use as a filter for fluorescence photography [19].

The IR filters, RG665, 715 and 780 (Figure 3g), behaved as expected, blocking short-wavelength light with cutoffs in the expected regions.

The Quaser filters are long-pass filters that cover a range of wavelengths from 420 to 600 nm (Figure 3h).

In summary, with regards to the transmission of the filters, the Schott GG420 behaved very similarly to Schott KV-418 in terms of cutoff wavelength and the rate of rising of the transmission curve. However, as discussed above, the Schott GG420 exhibited a high degree of fluorescence under UV light, which is an issue when using it as a filter for fluorescence imaging. Of the commercially available camera filters that were tested here, the Zeiss T* UV filter was the closest to the Schott KV-418, as it started to transmit at a similar wavelength (at around 405 nm), although its transmission did rise faster than the Schott KV-418, reaching 50% at around 411 nm vs. 418 nm for the Schott KV-418. The Baader UV/IR had a very similar wavelength for 50% transmission to the Schott KV-418 and GG420 and, like the Zeiss T* UV filter, also exhibited very low fluorescence under UV light.

**Figure 4:**
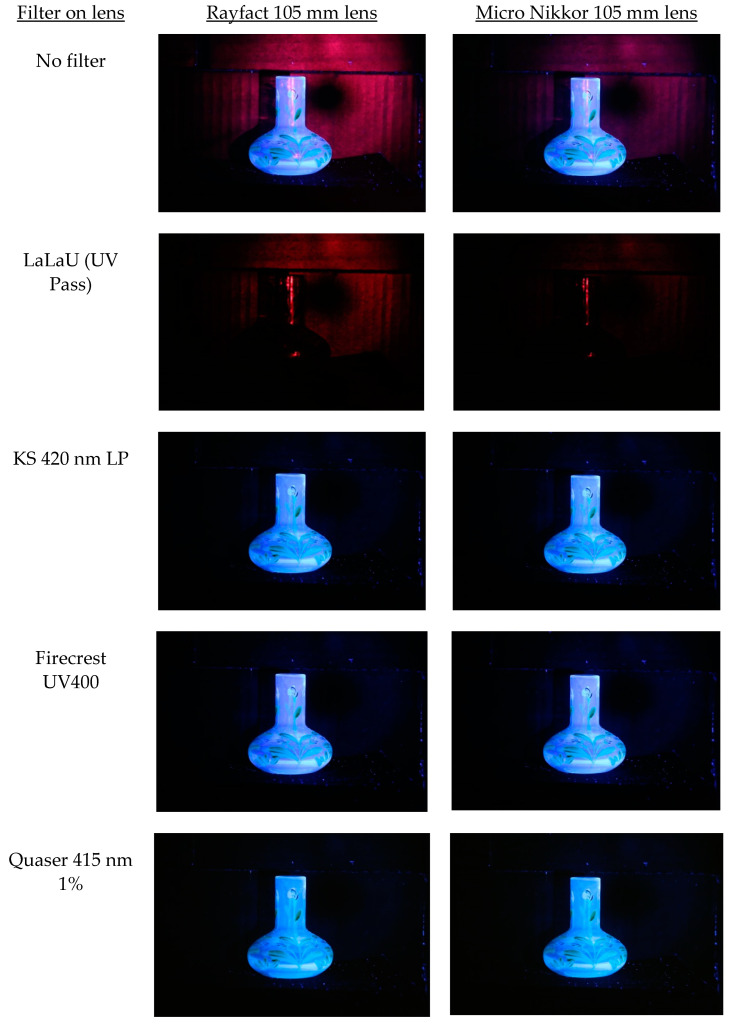
Images of test vase using two lenses (Rayfact 105 mm UV and Micro Nikkor 105 mm) with no filter on the lens, a UV pass filter (LaLa U) and three different UV blocking filters (KS 420 nm LP, Firecrest UV400 and Quaser 415 nm 1%). An unmodified Canon EOS 6D was used to capture the images.

In order to demonstrate the issues which can occur when using these types of filters, fluorescence imaging of a glass vase is shown in Figure 4. With no filter on the lens, the images show a mixture of fluorescence (the glass shows a strong blue fluorescence) but also evidence of specular reflection of UV. The specular reflection of the UV shows a strong red color and can be seen on the glass vase and the black background. The UV appears red due to the spectral response of the sensor in the UV [23]. While the camera was unmodified and contained filters that should be blocking UV and IR, these filters were not sufficient to block the specularly reflected UV, highlighting the need for using blocking filters when doing fluorescence imaging. The specular reflection of UV was stronger with the Rayfact 105 mm UV lens than with the Micro Nikkor 105 mm lens, as the Rayfact is more transparent to UV at 365 nm, showing how the choice of lens can also influence the imaging process. The specularly reflected UV component of the image can be seen when a UV pass filter that blocks visible and IR light (LaLa U) is placed on the lens. This prevents the fluorescence from being imaged while allowing the specularly reflected UV to be captured, which demonstrates that for the Canon EOS 6D used here, the internal filters that are present inside it were not blocking all the specularly reflected UV light. With two of the UV blocking filters (KS 420 nm LP and Quaser 415 nm 1%) attached, the specular reflection of UV is blocked, and the fluorescence is more clearly seen; however, a very slight red color cast is seen on the vase imaged with the Firecrest UV400, suggesting that it is not blocking the UV as effectively as the other two filters. In Figure 3a, the Firecrest 400 UV does seem to let in more of the longer wavelength UV light, which would account for this observation.

**Figure 5:**
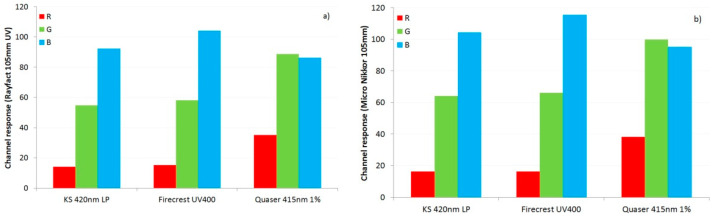
Red, green and blue channel response from an area of the black background in the RAW images for the three UV blocking filters for (**a**) the Rayfact 105 mm UV lens and (**b**) Micro Nikkor 105 mm lens.

In Figure 4, it is difficult to see the effects that the different UV blocking filters were having on the color balance of the images as a result of the fluorescence, as the effects are quite subtle (all images were captured using the same white balance settings). Isolation of a region of the low reflectance black background and assessment of the red, green and blue color channels of the original RAW images from the two lenses for the three fluorescence filters is shown in Figure 5.

As shown in Figure 5, with the KS 420 nm LP and Firecrest UV400 filters, the red, green and blue responses are very similar for both test lenses. However, with the Quaser 415 nm 1% filter, the red and green responses are much higher than with the other filters. The Quaser 415 nm 1% filter showed a strong yellow fluorescence under UV light (Figure 2), and it is likely that the increased red and green channel response seen for that filter is due to its yellow fluorescence. Overall, this demonstrates how both the transmission and fluorescence characteristics of a UV blocking filter can influence the color balance of the final image. This behavior is important for applications where color accuracy is important such as forensics, art conservation and dermatology [1,2,3,4,7,15,19].

While the phenomenon of UV-induced fluorescence of photographic filters is well known [19], relatively little has previously been published on quantifying how they fluoresce [20]. Five filters tested here were also reported in [20]. In the work presented here, those five filters ranked in the following order for the degree of fluorescence, with 3–4 orders of magnitude difference between the GG400 and RG715 filters:

GG400 > GG420 >> RG665 > RG780 > RG715

In the work presented in [20], these five filters ranked in the following order, again with 3–4 orders of magnitude between them:

GG420 > GG400 >> RG665 > RG780 > RG715

Both the work presented here and the results in [20] showed the GG400 and GG420 filters to have much higher fluorescence than the RG test filters, and both approaches ranked the low fluorescence RG filters in the same order. The highly fluorescent GG400 and GG420 filters were ranked differently between the work presented here and [20]. Possible reasons for this include the difference in the light source used (monochrome 365 nm in [20] compared to a broad band 320–400 nm light source here), and also, as mentioned in [20], there could be batch to batch variation in fluorescence for the glasses. Unfortunately, Schott KV-418 was not included in the work in [20]. Given the degree of correlation between the work presented here and that in [20], the author is confident that the described method worked as expected and is accurately able to compare the test filters.

While no longer manufactured by Schott, some suppliers were still offering KV filters during the preparation of this article [27], where the extended KV series of filters are described as being of very low fluorescence, making them useful for use as fluorescence barrier filters. It should also be noted that while researching for this article, one supplier was found to be advertising a filter called KV 418, although the transmission spectra for the filter advertised were different from the one supplied by Schott (and the one shown here). Therefore it is the author’s recommendation that anyone considering buying one of these filters should ask for actual transmission spectra and evidence of the lack of fluorescence before doing so. If the researcher does specifically need to locate Schott KV-418, KV filters are different from most photographic filters in terms of how they are constructed, which can assist in their identification. The edge of a KV filter shows a three-layer construction, with an obvious central layer being visible. In the Schott KV-418, this central layer is pale yellow. Moreover, the thickness of a KV filter should be 3.0 mm ± 0.2 mm, and the cutoff wavelength (in this case, 418 nm) is etched into to glass towards the edge of the filter. Some other filters have this type of layered construction, and that includes filters from Tiffen, so it should not be seen as a definitive method of identification; however, a Schott KV series filter has this three-layer construction. This method of construction may be one of the reasons why the Tiffen filters tested here exhibited low fluorescence, although that is purely a hypothesis at this stage.

For the measurements discussed here, the author used a 420 nm long-pass filter (described as the KS 420 nm LP) on the camera lens. This was chosen as the filter for use on the camera lens as it possessed very low fluorescence and good blocking of the UV. However, this filter came from a short production run and is no longer available from the supplier, and its thickness of 1.1 mm makes it very delicate. The primary aim of the work described here was to determine which filters are readily available, fluoresced similarly or less than Schott KV-418, and could therefore potentially be used instead of it. As such, while the KS 420 nm LP filter showed low fluorescence and good UV blocking, as it is not commercially available, it is not considered a recommended filter and is not discussed further.

During the preparation of this article, the author found that versions of some of the Schott OG and GG long-pass filters are available with coatings on them that are designed to reduce the degree of fluorescence under UV light and in sizes above the common 52 mm diameter. These filters were designed for use in the area of forensics photography [28]. Unfortunately, these filters were discovered after the testing discussed here was carried out, and therefore the author was unable to perform any independent testing of their fluorescence.

As can be seen in Table 1, the test filters have different thicknesses. This information was included, especially for the Schott filters, as the optical properties of the filters change as a function of thickness and as such thickness of a filter should be reported when discussing scientific imaging. The thickness has a direct effect on transmission through the filter, the effects of which can be seen in [29]. However, the effects of variations in thickness on fluorescence are less clear. In order for the fluorescence to occur as a result of the UV light source, then the incoming UV light must reach that part of the filter to initiate the fluorescence. With filters such as the 3.0 mm thick GG400 and GG420 tested here, which exhibit strong blocking of the UV, then it is likely that the fluorescence is being initiated close to the surface of the filter rather than throughout their entire thickness. It is, therefore, also likely that the degree of fluorescence observed for different thicknesses of those filters is not scaled directly as a function of filter thickness (for instance, a 1.0 mm thick version of the filter may not display 1/3 the fluorescence of a 3.0 mm thick version of the same filter). For filters with dichroic coatings, such as the Baader UV/IR and B + W UV/IR filter, the UV blocking would be expected to occur as a result of the coating, and therefore it is stopped (or at least drastically reduced) before it reaches the glass of the filter beneath the coating. Therefore filter thickness would not be expected to significantly impact the degree of fluorescence from these types of coated filters.

In [20], the authors discussed how fluorescence could vary between different batches of the same filter glass. In their testing, some filter glasses exhibited high batch-to-batch variability in their degree of fluorescence, while others were very low. The authors mentioned that fluorescence is a “side effect”, which is typically not specified in a datasheet, and therefore this would mean that it is not a release characteristic for the batch. Fluorescence is dependent on the impurities in the raw material of the glass melt, and due to small variations in the starting raw materials, it can fluctuate from batch to batch. While multiple batches of the different glasses tested here were not available to the author, two versions of one of the commercial photographic filters—the Tiffen Yellow 12—were tested. Both versions showed lower fluorescence than the Schott KV-418 filter; however, one version of it had twice the level of fluorescence of the other version, highlighting the need for the researcher to be able to determine how their own filters behave when designing an imaging system.

It is also worth mentioning the shape of the transmission curve for Schott KV-418 and some of the other filters, as shown in Figure 2. The KV-418 curve rises from around 405 nm and then continues to increase quite gently, passing 90% transmission at 436 nm before reaching 93% at around 450 nm. After reaching maximum transmission of around 93%, this then starts dropping at around 550 nm, before reaching 90% at 700 nm. The Zeiss T* UV, Firecrest UV400 and Baader UV/IR cut filters exhibit a much faster rise above the cutoff wavelength, reaching 95% transmission at around 435 nm and maintaining that high transmission to 700 nm and beyond. As a result of this, the Schott KV-418 would be expected to provide a slight green color cast to the images by removing some of the short-wavelength blue light, and slightly reducing the red light, when compared to some of the other filters. Correct white balancing of the image should be able to correct for this, and whether or not it is an issue depends on the particular application; however, it is something to be aware of when choosing a suitable filter.

Overall, when taking into account the level of fluorescence and the UV blocking of the filters, the Zeiss T* UV filter performed well and would be recommended for use where a Schott KV-418 is not available. The Zeiss T* UV had slightly lower fluorescence than the Schott KV-418, possessed good UV blocking, had a similar cutoff wavelength but reached 50% maximum transmission at a slightly shorter wavelength, had greater maximum transmission, is available in different sizes, and most importantly, is currently commercially available. The Baader UV/IR cut filter also performed well in terms of fluorescence and blocking of the UV; however, it is only available in limited sizes, which may limit its use. It was previously discussed as a potential filter for UV fluorescence [19]. If a slightly higher cutoff wavelength is required, then the Tiffen Yellow 12 filters both performed well and had low fluorescence and good UV blocking. Given that two different versions were tested and behaved very similarly, this suggests that batch-to-batch variability is low. Tiffen Yellow 8 had greater fluorescence than Schott KV-418 and also had a leak in the UV part of transmission spectra and would therefore not be recommended. The Firecrest UV400 filter showed low fluorescence but did not block all the long-wavelength parts of the UV. It does therefore have some potential for use as a UV blocking filter, depending on the spectral profile of the illuminant. The filters showing high fluorescence are not recommended for use given the possibility of their fluorescence from specularly reflected UV contaminating the final image.

Given that a technical method for assessing the filter fluorescence was discussed here, the limitations of the method and the approach should also be discussed. A very intense broad band light source was used to illuminate the test filters. This had a wide wavelength range of UV from 320 nm to 400 nm. As fluorescence is dependent on the illuminant wavelength [20], if the spectral characteristics of the researcher’s light source are not the same as the one used here, the fluorescence of the filter may be different. For instance, if a light-emitting diode (LED) light source is used, then than is likely to be a more tightly confined wavelength range. If a flash-based light source is being used, then that too gives a wide range of emitted light, more like the spectral signature shown here [30]. The degree of fluorescence of some of the filters was very low, and capturing that on a camera meant that high ISOs were needed, which can introduce digital noise into an image due to the increased sensitivity. It was the author’s experience that the camera used here (Canon EOS 6D) is capable of high ISO imaging with relatively low noise, and, as such, it is the author’s opinion that imaging at ISO6400 was an acceptable compromise to capture the images of the test filter behavior. The fluorescence color was assessed by eye either during the experiment or from the photographs. Unfortunately, it was not possible to measure the fluorescence spectra spectroscopically, as the setup used was not sufficiently sensitive. One ever-present issue when capturing fluorescence images is that of lint and fabric fibers present in dust [31]. If these fibers are from clothing, then they often fluoresce very strongly under the influence of UV light due to the presence of optical brighteners used in laundry detergents. The filters were directly cleaned before imaging was performed, and if any bright specs were observed, then the filter was re-cleaned and imaged again.

Obviously, the filters presented here represent a small proportion of the ones available to the researcher. How can the researcher repeat this method for themselves to determine whether the filters they wish to use fluoresce? The essence of the approach described here is to use a light source that produces known emission spectra. The filter fluorescence can then be assessed in a dark room using a camera with a suitable lens and a blocking filter present. In the work described here, a 200 W Xenon lamp was used, which was filtered using a Baader U-Venus filter to ensure only UV light hit the test filter. The lamp used, a Hamamatsu LC8, was sourced second-hand for around GBP 200, and the author used a Baader U-Venus filter that is normally used for photography or astronomy to filter that light. However, a cheaper approach would be to use a 365 nm LED torch such as a Convoy S2+ and to filter that with 2 mm Hoya U-340 glass to ensure that only UV is being emitted. Given the power of the LED-based lights, this is likely to produce a lower degree of fluorescence than the 200 W Xenon lamp used here, although longer exposure times would be needed for the imaging. In theory, any lens can be used, but the key would be to use a good UV blocking filter on the lens. The Zeiss T* UV filter identified here would meet the criteria both for UV blocking and low fluorescence and is available in a variety of sizes to suit different lenses, and as such, would be the recommendation of the author for this role, in addition to being used as a replacement for Schott KV-418. For the lens, a wide aperture (small maximum f stop number) would help with reducing exposure times for the imaging. The test filter should be placed inside a black enclosure to eliminate stray light. This can be prepared using cardboard, and the Semple Black 2.0 (or newer version 3.0) paint, as this was shown to have good UV absorption and low fluorescence [21]. It is still recommended that the experiments be carried out in a darkened room, though, as fluorescence imaging is extremely sensitive to ambient light. Filters to be imaged should be cleaned before testing, as should the inside of the enclosure they are being imaged in. Lint from clothing is a common component of dust, and optical brighteners used in laundry detergents mean that these fibers fluoresce strongly under UV. If the filter being tested or the enclosure itself has these present, then the image can quickly become contaminated by the light they emit. Moreover, as always, when using UV light, the safety of the researcher should be of primary concern, and the appropriate UV blocking safety glasses should be used [32,33].

## 4. Conclusions

Scientific photographic imaging is a complex process, and fluorescence imaging adds further factors which the visible light photographer does not have to deal with. However, with a relatively simple setup, it is possible to image and quantify one of those factors—photographic filter fluorescence under UV light.

A range of camera filters was tested for fluorescence on exposure to UV light, and their optical transmission spectra between 250 nm and 800 nm were determined. From the filters tested here, and based on the combination of low fluorescence, similar cutoff wavelength and commercial availability in a range of sizes, the Zeiss T* UV filter was determined to be the closest match to the no longer manufactured Schott KV-418 filter for use as a UV blocking filter when imaging fluorescence. The Baader UV/IR cut filter offered good blocking of the UV, combined with low fluorescence; however, its limited range of sizes may restrict its use in certain applications. The Firecrest UV400 filter also offered low fluorescence, although its slightly lower cutoff in the UV may restrict its use with some light sources. The need for the use of these types of filters in fluorescence photography was demonstrated using an object illuminated with UV.

A method for the researcher to assess the fluorescence of their own photographic filters was also described, enabling them to determine the suitability of their own equipment for their imaging needs.

## Figures and Tables

**Table 1:** Test filter degree of fluorescence normalized to ‘per second’, the observed fluorescence color and filter thickness.

Filter	Average Fluorescence s^−1^	Color of Fluorescence	Thickness
Baader UV/IR cut	15.3	No color	1.9 mm
B + W 486 UV/IR cut	12,048.3	Blue	2.0 mm
Firecrest UV400	17.4	No color	1.1 mm
Heliopan Yellow 5	30,886.6	Orange	1.9 mm
Hoya Y(K2)	14,689.0	Yellow/orange	2.2 mm
KS 420 nm LP	10.7	No color	1.1 mm
Quaser 415 nm 1%	41,525.1	Yellow	3.0 mm
Quaser 476 nm 1%	12,046.2	Orange	3.0 mm
Quaser 510 nm 1%	22,769.2	Yellow	3.0 mm
Quaser 549 nm 1%	431.3	Red/orange	3.0 mm
Quaser 593 nm 1%	2771.9	Orange	3.0 mm
Schott GG400	49,779.7	Yellow	3.0 mm
Schott GG420	38,689.6	Yellow	3.0 mm
Schott KV-418	127.0	Pale yellow	3.1 mm
Schott RG665	100.4	Pale red	3.0 mm
Schott RG715	11.0	Pale red	3.0 mm
Schott RG780	27.4	Pale red	3.0 mm
Tiffen Yellow 8	270.9	Yellow	2.7 mm
Tiffen Yellow 12, (version 1)	112.3	Yellow/orange	2.3 mm
Tiffen Yellow 12, (version 2)	51.2	Yellow/orange	2.3 mm
Zeiss T* UV	95.0	Pale orange	1.9 mm

## Data Availability

Data from this research are not available elsewhere. Please contact the author for more information if required.
